# Complex relationships between *Aedes* vectors, socio-economics and dengue transmission—Lessons learned from a case-control study in northeastern Thailand

**DOI:** 10.1371/journal.pntd.0008703

**Published:** 2020-10-01

**Authors:** Benedicte Fustec, Thipruethai Phanitchat, Mohammad Injamul Hoq, Sirinart Aromseree, Chamsai Pientong, Kesorn Thaewnongiew, Tipaya Ekalaksananan, Michael J. Bangs, Vincent Corbel, Neal Alexander, Hans J. Overgaard

**Affiliations:** 1 University of Montpellier, Montpellier, France; 2 Department of Microbiology, Faculty of Medicine, Khon Kaen University, Khon Kaen, Thailand; 3 Institut de Recherche pour le Developpement, Montpellier, France; 4 Department of Medical Entomology, Faculty of Tropical Medicine, Mahidol University, Bangkok, Thailand; 5 School of Public Health, Epidemiology and Social Medicine at the Institute of Medicine, University of Gothenburg, Gothenburg, Sweden; 6 HPV & EBV and Carcinogenesis Research Group, Khon Kaen University, Khon Kaen, Thailand; 7 Office of Diseases Prevention and Control, Region, Khon Kaen, Thailand; 8 Public Health & Malaria Control, PT Freeport Indonesia/International SOS, Mimika, Papua, Indonesia; 9 Department of Entomology, Faculty of Agriculture, Kasetsart University, Bangkok, Thailand; 10 MRC Tropical Epidemiology Group, London School of Hygiene and Tropical Medicine, London, United Kingdom; 11 Norwegian University of Life Sciences, Ås, Norway; Fundacao Oswaldo Cruz, BRAZIL

## Abstract

**Background/Objectives:**

Dengue fever is an important public health concern in most tropical and subtropical countries, and its prevention and control rest on vector surveillance and control. However, many aspects of dengue epidemiology remain unclear; in particular, the relationship between *Aedes* vector abundance and dengue transmission risk. This study aims to identify entomological and immunological indices capable of discriminating between dengue case and control (non-case) houses, based on the assessment of candidate indices, as well as individual and household characteristics, as potential risk factors for acquiring dengue infection.

**Methods:**

This prospective, hospital-based, case-control study was conducted in northeastern Thailand between June 2016 and August 2019. Immature and adult stage *Aedes* were collected at the houses of case and control patients, recruited from district hospitals, and at patients’ neighboring houses. Blood samples were tested by RDT and PCR to detect dengue cases, and were processed with the Nterm-34 kDa salivary peptide to measure the human immune response to *Aedes* bites. Socioeconomic status, and other individual and household characteristics were analyzed as potential risk factors for dengue.

**Results:**

Study findings showed complex relationships between entomological indices and dengue risk. The presence of DENV-infected *Aed*es at the patient house was associated with 4.2-fold higher odds of dengue. On the other hand, *Aedes* presence (irrespective of infectious status) in the patient’s house was negatively associated with dengue. In addition, the human immune response to *Aedes* bites, was higher in control than in case patients and *Aedes* adult abundance and immature indices were higher in control than in case houses at the household and the neighboring level. Multivariable analysis showed that children aged 10–14 years old and those aged 15–25 years old had respectively 4.5-fold and 2.9-fold higher odds of dengue infection than those older than 25 years.

**Conclusion:**

DENV infection in female *Aedes* at the house level was positively associated with dengue infection, while adult *Aedes* presence in the household was negatively associated. This study highlights the potential benefit of monitoring dengue viruses in *Aedes* vectors. Our findings suggest that monitoring the presence of DENV-infected *Aedes* mosquitoes could be a better indicator of dengue risk than the traditional immature entomological indices.

## Author summary

Dengue fever is a globally expanding arboviral disease, consisting of four distinct serotypes, transmitted primarily by synanthropic/peridomestic mosquitoes, *Aedes aegypti* and *Aedes albopictus*. Given the absence of specific treatment, and the incomplete protection provided by the currently available vaccine, vector surveillance and control remain the principal tool to prevent and control dengue transmission. However, vector surveillance through the monitoring of larval mosquito indices lacks consistency in addressing dengue risk. Surveillance based on pupal and adult stages is considered as more accurate to estimate dengue transmission risk, although monitoring is difficult to implement in routine. An alternative strategy is the use of the specific human antibody response to *Aedes* saliva to identify human exposure risk to *Aedes* bites. We conducted a hospital-based, case-control study in northeastern Thailand in order to identify risk factors for dengue infection using entomological and immunological indices, together with select individual and household characteristics. We found that people aged 10–25 years had significant higher odds of dengue than older adults (>25 years old). The presence of DENV-infected *Aedes* in the house was associated with 4.2-fold higher odds of dengue infection. Interestingly, *Aedes* adult abundance in the household was negatively associated with dengue revealing the complex role of *Aedes* density to dengue risk. This study highlights the potential benefit of monitoring dengue viruses in *Aedes* vectors to identify areas (“hot spots”) and people (“hot pops”) at higher risk of transmission.

## Introduction

Dengue fever is a globally expanding mosquito-borne disease which threatens half the world’s population [1]. Dengue virus (DENV) is transmitted by synanthropic *Aedes* mosquitoes, with *Aedes aegypti* (L.) typically being the primary vector [2], and *Aedes albopictus* (Skuse) a secondary one [3]. The Southeast Asia region accounts for more than half of the reported dengue cases worldwide [2, 4, 5]. Thailand typically records more than 20,000 cases each year, with all four DENV serotypes circulating and both vector species spread throughout the country [6]. Although dengue incidence is highly seasonal, outbreaks are difficult to predict [7, 8]. Dengue virus transmission is highly efficient and it is assumed that only a few vector mosquitoes are sufficient to ensure transmission [9]. *Aedes aegypti* is particularly well adapted to urbanized environments and is a strongly anthropophagic diurnal blood feeder [10–13]. The absence of specific treatments for dengue and the incomplete protection offered by the currently available vaccine [14, 15], underscores the importance of vector surveillance and management as the principal strategy for dengue prevention and control [7, 16].

In Thailand, dengue prevention and control are mainly based on hospital case reporting and vector surveillance and control that are carried out collaboratively between hospitals and the Offices of Disease Prevention and Control (ODPC). When a dengue case is reported from hospital, a Surveillance and Rapid Response Team (SSRT) is mandated to carry out insecticide space spray (‘fogging’) within 100 meters of the case house within 24 hours of notice in order to interrupt transmission [17]. The reorganization of disease control operations in Thailand resulted in 76 provincial administrations being aggregated into 22 regional ODPCs [18]. The seventh regional ODPC includes four provinces: Khon Kaen, Roi Et, Maha Sarakham, and Kalasin with a total population of around 5 million. Northeastern Thailand is the third largest region in the country with regards to population size and land area, with an economy mainly based on agriculture.

In most dengue-endemic countries, vector surveillance usually consists of monitoring *Aedes* immature (larvae and pupae) stages present in natural and artificial breeding sites (larval habitats) in and near houses [19–21]. Vector presence and density are estimated by standardized indices such as the Breteau Index (BI), Container Index (CI), House Index (HI), and the Pupae per Person Index (PPI) [21–23]. Entomological measures as thresholds have been proposed to assess and estimate risk for use as early warning systems to predict dengue outbreaks[19, 22, 24]. In Thailand, vector density thresholds to estimate risk of dengue outbreaks occurrence have been set at HI>10, BI>50 and CI>1 [25]. Additionally, vector control interventions are implemented to reduce vector abundance and prevent dengue transmission. However, numerous studies have failed to clearly link entomological indices to the risk of dengue transmission [7, 24, 26, 27]. Indeed, the larval stages (four successive instars) typically suffer high mortality during development to pupal stage, thus indices based only on their presence are generally poor indicators of the eventual adult vector density. Pupal indices (a stage with very low mortality) were proposed as a more accurate determination of actual adult production; however, pupal collections are far more challenging and time consuming to carry out [26, 28]. Adult collections can be performed via several devices such as gravitraps, sticky traps, baited mechanical traps, and mouth or mechanical aspirators, but they only provide an imprecise estimation of the true vector density and do not reflect human-vector exposure.

Entomological collections for target *Aedes* species, of all kinds, are labor- and time-consuming, expensive, and contingent on access to the house being granted. However, estimating the human immune response to *Aedes* bites as a surrogate measure of bite exposure (intensity) might be less labor-intensive and more informative of relative “vector attack” over time [29]. Upon initiating the blood feeding process, salivary gland proteins injected at the bite site induce a species-specific immune response by the host [30, 31]. These specific antibodies (against salivary proteins) have shown promising to measure seasonal variation of human exposure to mosquito bites [32–37] and to assess the effectiveness (i.e., reduction in biting) of vector control interventions [38].

The current study aims to identify risk factors for dengue transmission across four provinces in northeastern Thailand by comparing individuals with and without dengue in terms of i) their immune response to *Aedes* bites, ii) the presence and abundance of immature and adult *Aedes* in and close proximity around their houses, and iii) their individual and household characteristics. The first objective was to assess the accuracy of entomological and immunological indices to discriminate dengue positive and dengue negative households. We hypothesize that there will be more adult *Aedes* mosquitoes and a higher level of immune response to *Aedes* exposure (salivary proteins) in households with a recent dengue case compared to control (non-case) houses. The second objective was to assess whether socio-economics, household characteristics and entomological and immunological indices can be accurate predictors of dengue transmission risk.

## Materials and methods

### Study settings

**Fig 1:**
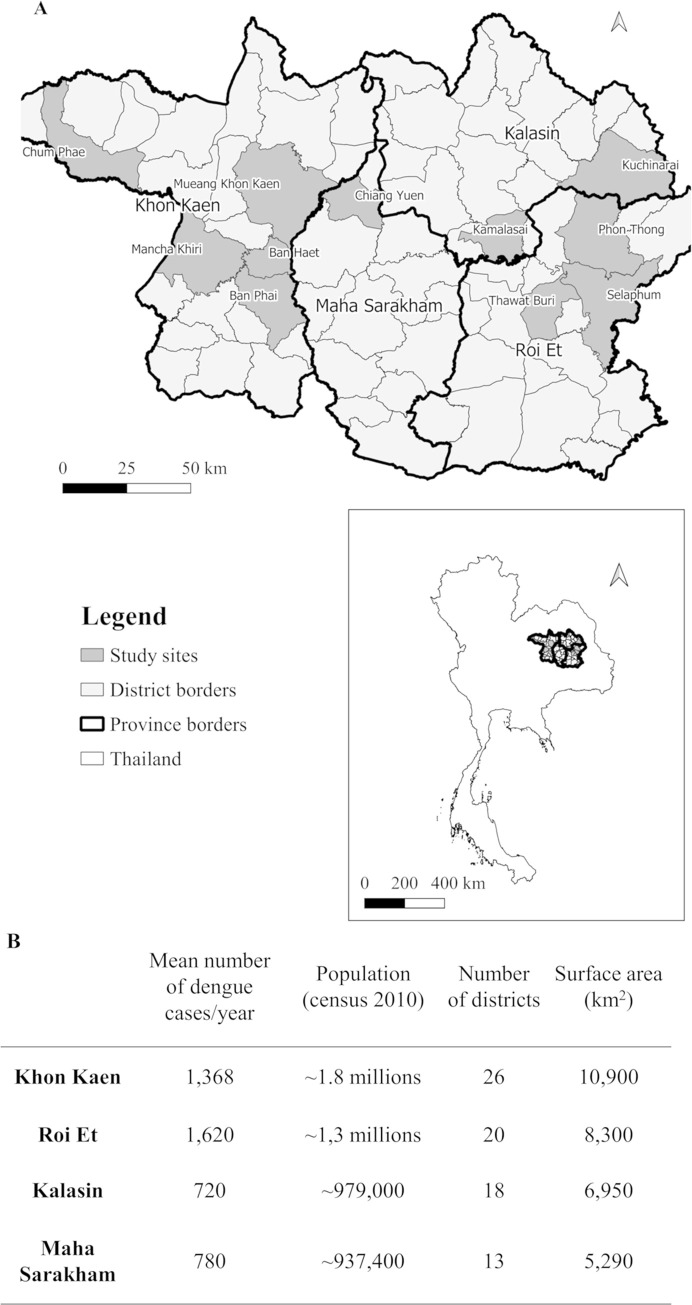
Map and characteristics of study sites of the case-control study in northeastern Thailand. **A:** Location of four provinces and study districts in northeastern Thailand included in the case-control study. Map of study sites was built using QGis 3.10 software and shapefiles were obtained from the Humanitarian Data Exchange project [41] under the Creative Commons Attribution International 4.0 license (CC BY 4.0). **B:** Study area characteristics, population and average number of dengue cases per year from 2005–2019 [39].

This hospital-based case-control study was carried out in four provinces in northeastern Thailand (Fig 1) between June 2016 and August 2019. Ten district hospitals were included: Mancha Khiri, Chum Phae, Ban Phai, and Ban Haet districts in Khon Kaen Province; Selaphum, Phon Thong, Thawatburi districts in Roi Et Province; Kamalasai and Kuchinarai districts in Kalasin Province; and Chiang Yuen district in Maha Sarakham Province. Additionally, nine sub-district hospitals in Khon Kaen Muang district (Khon Kaen Province) were included. The four provinces cover approximately 31,440 km^2^ with around 5 million inhabitants. Khon Kaen, Roi Et, Kalasin and Maha Sarakham provinces are divided in 26, 20, 18 and 13 districts, respectively (Fig 1). Over the previous 15 years, the region reported in average 4,488 dengue cases annually [39, 40]. A case-control design was chosen because it allowed the investigation of several risk factors concomitantly, it is effective for diseases with low incidence, and requires relatively, few study subjects.

### Sample size

The study sample size was calculated using the unmatched case-control study module of OpenEpi, version 3 [42] with 90% power based on data from Thomas et al. [43]. Assuming a difference in DENV-infected female *Aedes* mosquitoes collected between dengue positive and dengue negative households, with an exposure of 10% of DENV-infected *Aedes* in the exposed group, and 1% of DENV-infected *Aedes* in the control group, the significance level was set at 5% (two-sided) and the ratio of control to case at 1. The result was a target sample size of 322 patients. To allow for a 15% loss at the household questionnaire stage, we increased the final sample to 370.

### Patient recruitment

Patients presenting with dengue-like symptoms were recruited from the participating hospitals. Regarding Thai health services, public hospitals generally serve the communities in the districts and sub-districts in which they are located. Eligible patients with potential dengue infections were recruited based on presence of fever (≥38°C), no recent travel history during the previous 7 days, and being older than five years-of-age.

### Blood collections

**Fig 2:**
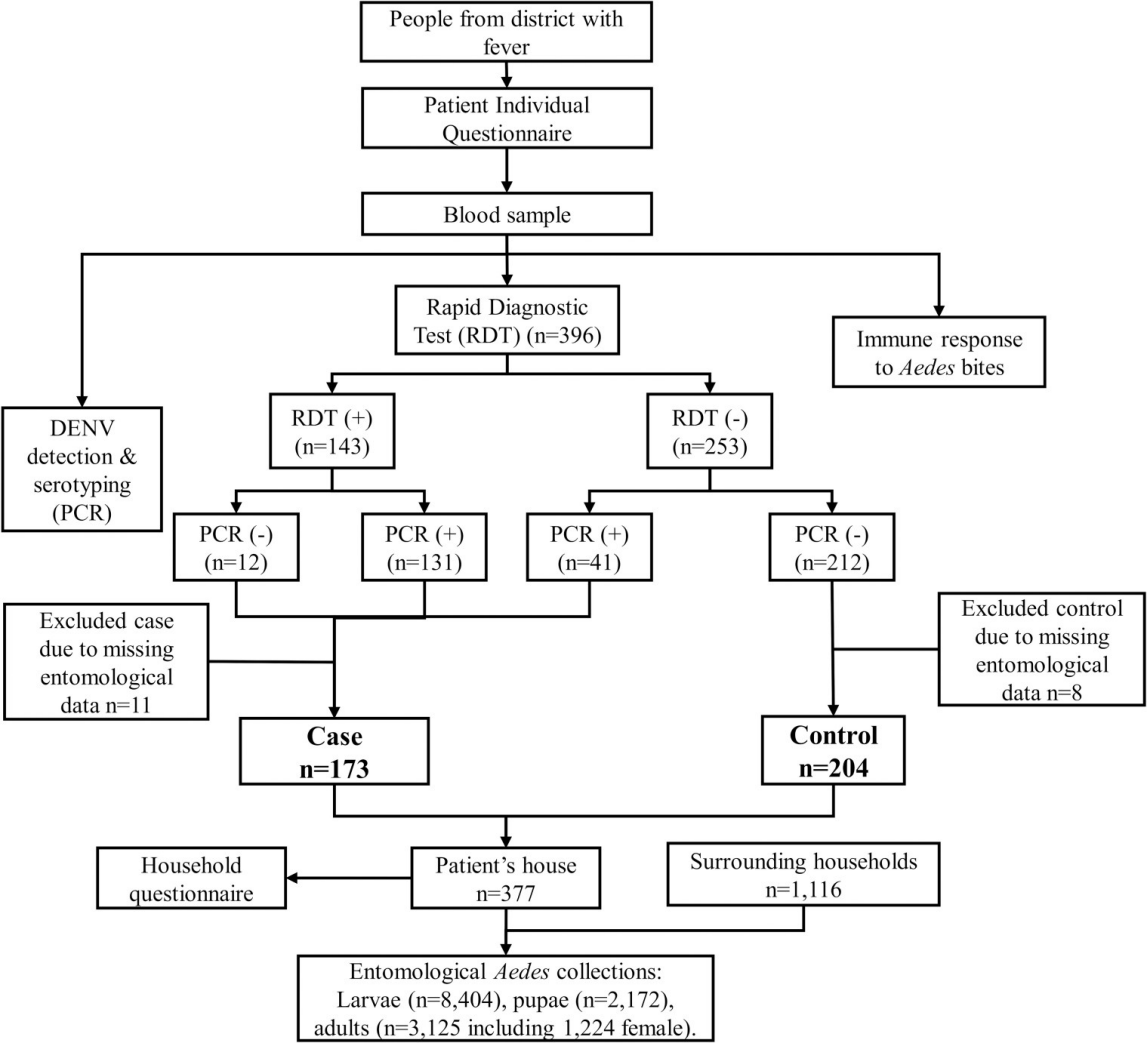
Flow diagram of case-control study design.

A total of 6 mL of venous blood was drawn from each participant for the following three purposes (Fig 2):

1. Detect dengue non-structural protein 1 (NS1) and IgM / IgG antibodies using a Rapid Diagnostic Test (RDT) (SD BIOLINE Dengue Duo, Standard Diagnostics, Korea).
2. Determine the immune response to *Aedes* bites using two blood drops (approximately 75μL each) collected on protein saver cards 903 (Whatman, UK).
3. Confirm dengue infection by reverse transcription polymerase chain reaction (RT-PCR) (described below) and distinguish serotypes (not presented here) using 5.7 mL whole blood collected in heparin or EDTA tubes.

### DENV confirmation in human samples and case definition

RNA was extracted from patients’ blood for DENV screening, confirmation and serotyping by RT-PCR as described previously [44] and adapted to conventional PCR. According to the course of dengue illness, viremia usually drops after few days of fever, while antibody response is triggered within few days after the beginning of dengue symptoms [2]. Therefore, a positive sample for NS1 and/or IgM by RDT and/or positive for DENV by PCR was recorded a dengue case. A participant who was negative for both RDT and PCR or IgG-positive only was recorded a control (Fig 2). Hence the controls were selected on the basis of having an “imitation” disease with similar symptoms (e.g., fever) to dengue [45], a design method also known as ‘test-negative’ [46].

### Individual characteristics

A questionnaire was used to collect information about each individual study case (positive and control). Patients were stratified into four age groups: 5–9 years-old; 10–14 years-old; 15–25 years-old; and > 25 years-old. History of previous dengue infections and vaccinations were recorded. Patients were asked about their main activities during weekdays and weekends (e.g., at home; at work away from home; at school; farming; other), as well as their typical resting/sleeping locations and habits (e.g., primarily indoor, outdoor, or equally indoor and outdoor). Travel history outside the resident district within the last three months was recorded and used as a binary variable.

### Household characteristics

A questionnaire was used to collect data on house characteristics and socio-economic status, including monthly household income, possession of certain assets (e.g., TV, air conditioner, car, or motorbike), and source of drinking and non-drinking water. Observations on the house included the number of rooms, wall and ceiling construction material, and presence or absence of eaves gaps. Housing was differentiated between those having a family living on one or two floors; other types of living conditions, such as apartments, townhouses, or multiple families living in separate houses grouped together. Mosquito control methods used in the household were divided as follows: (1) larval control, (2) adult mosquito control, (3) both the preceding, and (4) no control. The Premise Index was estimated based on the general condition of the house, the surrounding yard area and degree of shade [47].

### Entomological collections

Mosquito collections were systematically conducted in each patient house and in each of four surrounding houses. The total number of containers and those containing water were recorded at each household. A maximum of 20 third or fourth stage larval instars and all pupae were collected per container. Immature *Aedes* were identified to species using morphological keys [48, 49] and sex was determined for adults. Adult mosquitoes were collected using a battery-powered mechanical aspirator for 15 min indoors and 15 min outdoors in close proximity to house. Adults were identified to species and stored individually in 1.5mL micro-centrifuge tubes at -20°C until further analysis.

### DENV detection in *Aedes* mosquito samples

Female *Aedes* were separated and labelled by location (indoors/outdoors; patient house/ surrounding house). Up to 15 adult female mosquito abdomens were pooled for RNA extraction and DENV detection. Retained head-thorax sections corresponding to positive pools were individually screened for DENV and serotyping by qRT-PCR using the protocol of Lanciotti et al. [50] with minor modifications to perform it in real-time.

### Mosquito Exposure Index (MEI)

*Aedes*-specific immune response was evaluated in each case and control patient from dry blood spots by an indirect Enzyme-Linked Immunosorbent Assay (ELISA) using the Nterm-34kDa salivary peptide (Genepep, St Jean de Vedas, France), an established marker of human exposure to *Aedes* salivary gland proteins [38, 51, 52]. Blood samples collected on filter paper were cut by a one cm diameter hole punch. Blood spots were eluted in 400μL Phosphate Buffer Saline (PBS)-0.1% Tween for 24h hours at 4°C before removing the filter paper. Eluates were stored at -20°C until further processing. Preliminary assays were conducted to adapt the protocol to the human population living in the study areas using individuals exposed and unexposed to *Aedes* mosquitoes (see below). Briefly, the salivary peptide was coated at 20μg/mL for 150 min at 37°C into Maxisorp plates (Nunc, Roskilde, Denmark). After washing with a solution of demineralized water plus 0.1% of Tween detergent, the protein-free blocking buffer (Pierce, Thermo Fisher, USA) was incubated for 1h at room temperature. Blood eluates diluted at 1:160 in PBS+1% Tween were incubated overnight at 4°C. Biotin-conjugated goat anti-human IgG (Invitrogen, Thermo Scientific, USA) was incubated at 1:6000 dilution for 1h30 at 37°C. Streptavidin HRP-conjugate was incubated for 1h at 37°C at 1:4000 dilution. Colorimetric reaction was performed using ABTS buffer (2,2’-azino-bis (3-ethylbenzthiazoline 6-sulfonic acid) di-ammonium) + 0.003% H_2_O_2,_ and absorbance (optical density, OD) measured after 120 min at 405nm with Sunrise spectrophotometer (Tecan, Switzerland). Samples were assayed in duplicate and in a blank well (no antigen) to measure individual background and antibody response (ΔOD) expressed as:

$$\Delta OD=\mathrm{mean}\;{\mathrm{OD}}_{\mathrm{Ag}+}‐{\mathrm{OD}}_{\mathrm{Ag}‐}$$ (1)

To quantify the non-specific immune reactions and calculate the immune threshold, anti-Nterm-34kDa IgG response was assayed from dried blood in individuals with no known exposure history to *Ae*. *aegypti* (i.e., blood samples from northern France collected between January and March 2016 to 2018, and Western Australia in October 2016). Specific immune threshold (TR) was defined as follows:

$$TR=\Delta {\mathrm{OD}}_{\mathrm{unexposed}\;\mathrm{individuals}}+3\;SD_{\mathrm{unexposed}\;\mathrm{individuals}}$$ (2)

This value was calculated as 0.45. The MEI is the sample-specific immune response to the salivary peptide defined as:

MEI=ΔOD−TR. (3)

MEI was categorized into three classes: low, medium, and high responder. Samples with an ΔOD below the 0.45 TR, and therefore with a negative MEI value, were categorized as non-responders.

### Entomological indices

Entomological indices in patients’ houses were distinguished from those at the neighborhood level (i.e. patient’s house + four surrounding houses, S1 Table). At the patient house level, the Container Index (CI) was calculated as the proportion of containers positive for immature *Aedes* among wet containers inspected. The Pupae per House Index (PHI) and the Pupae per Person Index (PPI) were calculated as the total number of pupae collected per house and the total number of pupae per person living in the patient’s house, respectively. The female adult *Aedes* Index (AI) and the female *Aedes* indoor Index (AI_in) represent the number of female adult *Aedes* collected both indoors and outdoors and those collected only indoors, respectively. The female *Aedes* infected Index (AI+) represent the proportion of all female sampled mosquitoes infected with DENV. At the neighborhood level, the House Index (HI) was calculated as the proportion of houses with immature *Aedes* and the Breteau Index (BI) as the number of *Aedes*-positive containers per 100 houses. The neighborhood Container Index (CI_n_), Pupae per House Index (PHI_n_), female *Aedes* Index (AI_n_), female Adult indoor Index (AI_n__in), and female *Aedes* infected Index (AI_n_+) were calculated the same as described above, but at the neighborhood level.

### Data analysis

Data analysis used R 3.5.1 software with the MASS, glm, and Rcmdr packages [53, 54]. Figures were designed using ggplot2 and ggpbur packages [55]. Map of study sites was built using QGis 3.10 software and shapefiles were obtained from the Humanitarian Data Exchange project CC-BY 4.0 [41]. Distribution of indices was visualized by kernel density estimate. Vector control measures, household observations and Premise Index are categorical variables. The study population was analyzed with descriptive statistics, and individuals’ information and household characteristics were analyzed with the dengue case occurrence as categorical variables using univariable logistic regression. The socio-economic status (SES) of each patient was calculated as a score based on the household questionnaire (e.g., assets, income) using principal component analysis [56]. A total of 16 items of durable household assets were used as proxies to estimate wealth status (S2 Table). The first principal component explained 17% of the variance. Based on this analysis, patients were categorized by tertiles of the first principal component in ‘wealth’ groups (high, intermediate, and low).

Univariable binomial logistic regression was performed between each entomological and immunological index and dengue case/control status. Multivariable logistic regression was performed using all variables (i.e. individual characteristics, house characteristics, SES, entomological and immunological indices) with a statistically significant association (p<0.1) with case/control status on the univariable analysis. Only individuals with complete data for the variables of interest were kept for the multivariable analysis. Because of the overdispersion of the distributions of the entomological indices, they were transformed from continuous to categorical data of two groups: the null group (index value = 0) and the positive values (index value > 0). Model selection was based on backward/forward Akaike Information Criterion (AIC) selection. All variables were first included in the model and the selection was made by removing variables and/or then adding them (backward/forward selection). At each step, the AIC was calculated and the selected model was the one with the lowest AIC. Wald confidence intervals (95% CI) were calculated. Potential confounding variables of most interest were those which were plausibly associated with both entomological indices and risk of dengue, in particular socio-economic status and travel history.

### Ethical statement

This study was approved from the Khon Kaen University Ethics Committee (KKUEC, project number HE591099), the London School of Hygiene and Tropical Medicine Ethical Committee (LSHTM Ethics, project number 10534), and the Norwegian Regional Committees for Medical and Health Research Ethics (REC, no. 2016/357). Each patient was fully informed about the study and, if agreeing to participate, provided signed informed consent. Patients 13–17 years old signed assent forms and their parents/guardians signed informed consent. Parents/guardians of patients 5–12 years old signed consent forms on the patient’s behalf. For participating neighboring households, information about the study was given and signed consent for entomological collections was obtained before beginning sampling.

## Results

### Dengue cases, individual and household characteristics of the population

All 396 patients informed about the study agreed to participate and were recruited. Some were excluded from the analysis because of missing entomological and household data, mostly because of limited capacity to follow-up multiple patients presenting at a facility on the same day (Fig 2). A total of 377 patients with complete entomological data were included in the final analysis, comprising 173 dengue cases and 204 controls (0.85 case/control ratio). The participant ages ranged from 5 to 76 years with 190 (48%) females represented (Table 1). Almost half of the dengue cases were between 10 and 14 years of age resulting in 4.28-fold higher odds for dengue infection than people aged greater than 25 years old (p<0.001). Similarly, individuals aged between 15 and 25 years of age had 3.23-fold higher odds for dengue than individuals above 25 years (p<0.001). The majority (60.4%) of the dengue case patients reported having lived in the respective district for more than ten years compared to 46% of the controls, yet there was no difference between the length of stay in the area and dengue risk (p = 0.200, p = 0.356 and p = 0.975 for a stay between 1 and 5 years, between 5 and 10 years and more than 10 years, respectively). Most of the study participants spent their time either at school or at home during the weekdays resulting in a lower odds of dengue for individuals working away from home or those at school compared to the people staying at home (OR: 0.48, 95% CI: 0.24–0.94, p = 0.033 and OR: 0.60, 95% CI: 0.37–0.97, p = 0.035, respectively). Working partly indoors and outdoors was associated with lower odds of dengue (p = 0.045) compared to working outdoors only. Although not statistically significant, there was a tendency for those working only indoors to have higher odds for dengue (p = 0.085). Travel outside the district in the previous three months was associated with lower odds of infection (p = 0.031).

**Table 1:** Individual and household characteristics and their associations with dengue fever cases in northeastern Thailand, June 2016 and July 2019. Odds ratios (OR), obtained by logistic univariable regression, in bold text are significant (p<0.05). Missing data by individual not included in the analysis.

		Case (n = 173)		Control (n = 204)		Total (n = 377)		OR (95% CI)	p-value
		n	(%)	n	(%)	n	(%)		
**Province**	Roi Et	45	(26.0)	47	(23.4)	92	(24.4)	Reference	0.835
	Khon Kaen	40	(23.1)	86	(42.2)	126	(33.4)	**0.49** (0.27–0.84)	0.011
	Maha Sarakham	54	(31.2)	49	(24.0)	103	(27.3)	1.15 (0.65–2.02)	0.624
	Kalasin	34	(19.7)	22	(10.8)	56	(14.9)	1.61 (0.82–3.16)	0.164
**Gender**	Male	95	(54.9)	101	(49.5)	196	(5.20)	Reference	0.668
	Female	78	(45.1)	103	(50.5)	181	(4.80)	0.79 (0.53–1.19)	0.274
**Age groups**	More than 25 years old	21	(12.1)	55	(27.0)	76	(20.2)	Reference	<0.001
	15 to 25 years old	42	(24.3)	33	(16.2)	75	(19.9)	**3.23** (1.64–6.36)	<0.001
	10 to 14 years old	85	(49.1)	52	(25.5)	137	(36.3)	**4.28** (2.33–7.88)	<0.001
	5 to 9 years old	25	(14.5)	64	(31.4)	89	(23.6)	1.02 (0.51–2.02)	0.948
**Lived in district**	Less than 1 year	7	(4.0.5)	6	(2.94)	13	(3.45)	Reference	0.782
	Between 1 and 5 years	16	(9.25)	31	(15.2)	47	(12.5)	0.44 (0.12–1.53)	0.200
	Between 5 and 10 years	44	(25.4)	65	(31.9)	109	(28.9)	0.58 (0.18–1.84)	0.356
	More than 10 years	102	(60.0)	88	(43.1)	190	(50.4)	0.98 (0.32–3.03)	0.975
	(Missing)	4	(2.31)	14	(6.86)	18	(4.77)	-	-
**Dengue diagnosed before**	No	138	(79.8)	138	(67.7)	276	(73.2)	Reference	1
	Yes, this year	11	(6.36)	14	(6.86)	25	(6.63)	0.78 (0.34–1.79)	0.566
	Yes, last year	1	(0.58)	7	(3.43)	8	(2.12)	0.14 (0.01–1.18)	0.071
	Yes, before last year	19	(11.0)	32	(15.7)	51	(13.5)	0.59 (0.32–1.10)	0.097
	(Missing)	4	(2.31)	13	(6.37)	17	(4.51)	-	-
**Spend week days**	At home	60	(34.7)	44	(21.6)	104	(27.6)	Reference	0.118
	At work away from home	21	(12.1)	32	(15.7)	53	(14.1)	**0.48** (0.24–0.94)	0.033
	At school/college/university	87	(50.3)	106	(52.0)	193	(51.2)	**0.60** (0.37–0.97)	0.035
	At farm	0	(0.00)	2	(0.98)	2	(0.53)	-	0.981
	Other	1	(0.58)	3	(1.47)	4	(1.06)	0.24 (0.01–1.98)	0.229
	(Missing)	4	(2.31)	17	(8.33)	21	(5.57)	-	-
**Spend week ends**	At home	148	(85.6)	148	(72.6)	296	(78.5)	0.99 (0.79–1.25)	0.954
	At work away from home	14	(8.09)	23	(11.3)	37	(9.81)	0.61 (0.30–1.24)	0.172
	At school/college/university	3	(1.73)	6	(2.94)	9	(2.39)	0.50 (0.12–2.05)	0.338
	At farm	1	(0.58)	3	(1.47)	4	(1.06)	0.33 (0.03–3.26)	0.347
	Other	3	(1.73)	4	(1.96)	7	(1.86)	0.76 (0.17–3.43)	0.716
	(Missing)	4	(2.31)	20	(9.80)	24	(6.37)	-	-
**Location of workplace**	Outdoors	54	(31.2)	59	(28.9)	113	(30.0)	Reference	0.638
	Indoors	76	(43.9)	53	(26.0)	129	(34.2)	1.56 (0.94–2.60)	0.084
	Both indoors and outdoors	38	(22.0)	72	(35.3)	110	(29.2)	**0.57** (0.34–0.99)	0.045
	(Missing)	5	(2.89)	20	(9.80)	25	(6.63)	-	-
**Travel within the previous 3 months**	No	156	(90.2)	162	(79.4)	318	(84.4)	Reference	0.695
	Yes	13	(7.51)	29	(14.2)	42	(11.1)	**0.46** (0.23–0.93)	0.031
	(Missing)	4	(2.31)	13	(6.37)	17	(4.51)	-	-
**Socio-economic status**	High	54	(31.2)	64	(31.4)	118	(31.3)	Reference	0.358
	Intermediate	50	(28.9)	69	(33.8)	119	(31.6)	0.61 (0.36–1.02)	0.060
	Low	64	(37.0)	54	(26.5)	118	(31.3)	0.71 (0.43–1.19)	0.194
	(Missing)	5	(2.89)	17	(8.33)	23	(5.84)	-	-
**Household type**	One family, one floor	47	(27.2)	79	(38.7)	126	(33.4)	Reference	0.005
	One family, two floors	111	(64.2)	97	(47.6)	208	(55.2)	**1.92** (1.22–3.02)	0.005
	Others	10	(5.78)	11	(5.39)	21	(5.57)	1.52 (0.60–3.87)	0.371
	(Missing)	5	(2.89)	17	(8.33)	22	(5.84)	-	-
**Wall spray**	No	127	(73.4)	117	(57.4)	244	(64.7)	Reference	0.565
	Yes	41	(23.7)	70	(34.3)	111	(29.4)	**0.54** (0.35–0.87)	0.010
	(Missing)	5	(2.89)	17	(8.33)	22	(5.84)	-	-
**Eaves gaps**	No	112	(64.7)	97	(47.6)	209	(55.4)	Reference	0.334
	Yes	56	(32.4)	90	(44.1)	146	(38.7)	**0.55** (0.36–0.84)	0.006
	(Missing)	5	(2.89)	17	(8.33)	22	(5.84)	-	-
**Vector control**	No	20	(11.6)	18	(8.82)	38	(10.1)	Reference	0.873
	Yes, against larvae	51	(29.5)	34	(16.7)	85	(22.6)	1.45 (0.56–1.97)	0.337
	Yes, against adult mosquito	28	(16.2)	11	(5.39)	39	(10.3)	2.41 (0.95–6.18)	0.065
	Yes, against both adult and larvae	69	(39.9)	124	(60.8)	193	(51.2)	0.52 (0.26–1.05)	0.068
	(Missing)	5	(2.89)	17	(8.33)	22	(5.84)	-	-

Although there was no strong evidence of dengue transmission risk associated with SES, certain physical house characteristics were relevant. Living in a single family, two-floor house had increased odds compared to living in a single-floor house, while the presence of eaves gaps had lower odds than house lacking them (Table 1). The majority of households (80–90%) used some kind of vector control method(s), but these were not significantly associated with dengue risk (p>0.06). In particular, adult mosquito control was more often used in case houses and was indicative of a higher odds of dengue (OR: 2.41, 95% CI: 0.95–6.18, p = 0.065), while a combination of larval and adult controls was more common in control houses, which showed a lower odds than houses using no vector control (OR: 0.52, 95% CI: 0.26–1.05, p = 0.068). Furthermore, insecticide applications to indoor wall surfaces (performed by vector control unit staff or private companies for dengue or pest control) was more common among controls than in the case group resulting in a lower odds of dengue in houses with sprayed walls in the last 12 months (OR: 0.54, 95%CI: 0.35–0.87, p = 0.010).

### Mosquito exposure index

**Fig 3:**
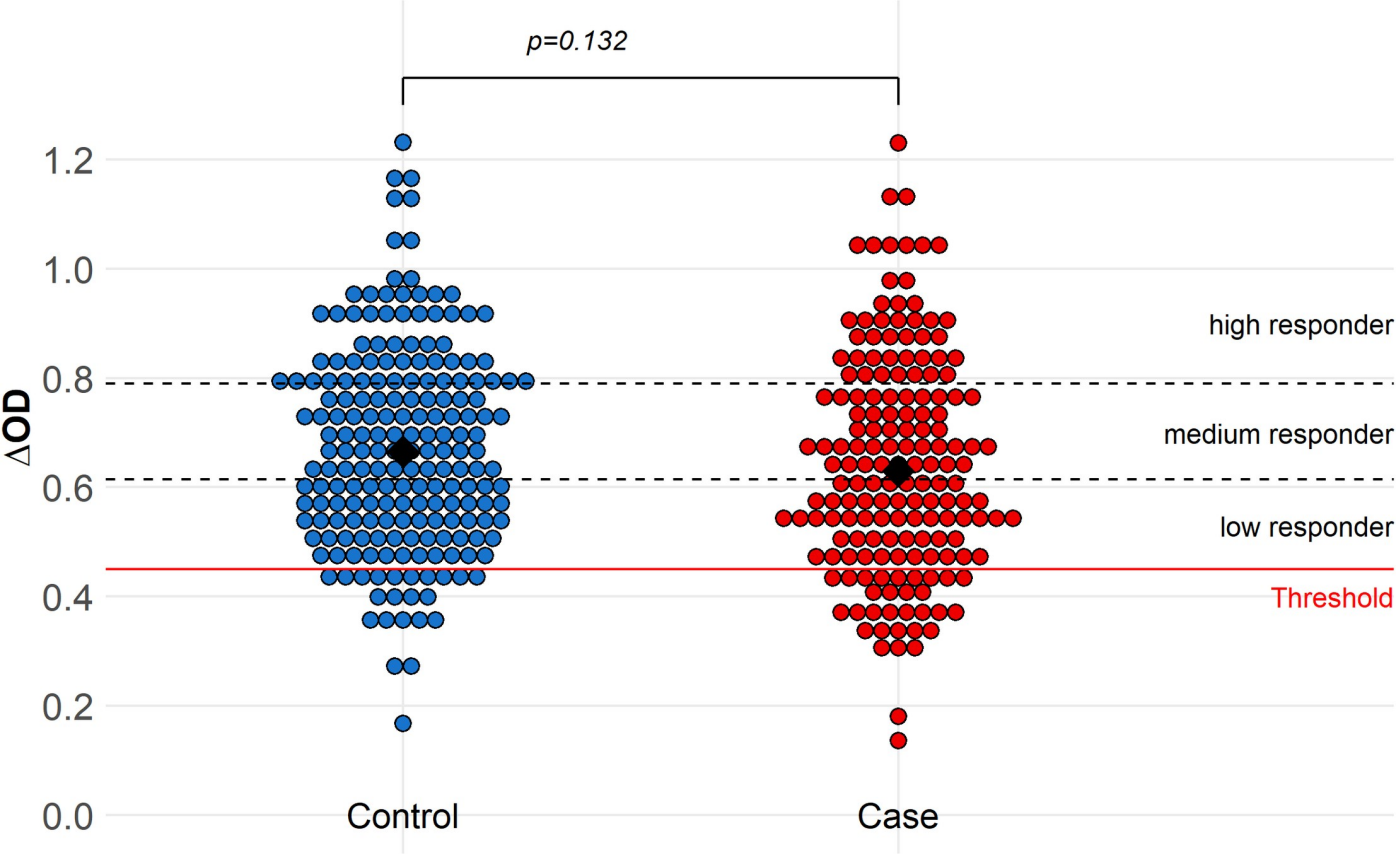
Immune response to *Aedes* saliva (ΔOD) in dengue case and control patients. The black diamonds represent the response medians. The dashed lines represent the limits of each group of intensity of response. The red line at 0.45 indicates the specific immune threshold TR defined from individuals not exposed to *Ae aegypti*.

Only 10% (n = 37 of 368) of the tested individuals (cases and controls) were non-responders to the *Aedes* Nterm-34kDa salivary biomarker as their specific immune response was below the immune threshold TR (Fig 3). There was not significant difference in antibody response to *Aedes* salivary biomarker between case and control. Although not significant, being a medium or high responder to mosquito salivary antigens, surprisingly, tended to be negatively associated with dengue risk relative to non-responders (OR = 0.51, 95% CI: 0.24–1.10, p = 0.08, and OR = 0.50, 95%CI: 0.23–1.07, p = 0.07) (Table 2).

**Table 2:** Immunological and entomological indices and their associations with dengue fever cases in northeastern Thailand, June 2016 and June 2019. Odds ratios (OR) obtained by logistic univariable regression, and confidence intervals (95% CI) by Wald’s statistics. Odds ratios in bold are significant (p<0.05).

		Case%	Control%	OR	95% CI	p-values
		(n = 173)	(n = 204)			
**Individual level**						
**MEI**	Non-responder	12.7	7.35	Reference		
	Low responder	31.2	28.4	0.63	[0.30–1.35]	0.237
	Medium responder	26.6	29.9	0.51	[0.24–1.10]	0.086
	High responder	27.2	31.9	0.50	[0.23–1.07]	0.073
	(Not determined)	2.31	2.45	-	-	-
**House level**						
**CI (%) (mean)**		29.1	37.3	**1.00**	[0.99–1.00]	0.044
***Aedes* Index (AI)**	0	61.3	48.5	Reference		
	>0	38.7	51.5	**0.59**	[0.39–0.89]	0.012
***Aedes* Index indoor (AI_in)**	0	67.6	52.9	Reference		
	>0	32.4	47.1	**0.53**	[0.35–0.81]	0.003
***Aedes* Index infected (AI+)**	0	91.9	96.6	Reference		
	>0	8.09	3.43	2.48	[0.97–6.28]	0.056
**Pupae per House Index (PHI)**	0	69.9	66.2	Reference		
	>0	30.1	33.8	0.83	[0.54–1.28]	0.397
**Pupae per Person Index (PPI)**	0	72.3	71.6	Reference		
	>0	27.7	28.4	0.95	[0.61–1.49]	0.824
**Neighborhood level**						
**BI** (mean)		68.6	93.4	**0.99**	[0.99–1.00]	<0.001
**HI (%)** (mean)		47.9	58.3	**0.99**	[0.98–1.00]	0.002
**CI**_**n**_ **(%)** (mean)		29.2	41.7	**0.99**	[0.98–1.00]	<0.001
***Aedes* Index (AI**_**n**_**)**	0	24.9	22.6	Reference		
	>0	75.1	77.4	0.87	[0.54–1.41]	0.581
***Aedes* Index indoor (AI**_**n**_**_in)**	0	29.5	26.5	Reference		
	>0	70.5	73.5	0.86	[0.54–1.34]	0.498
***Aedes* Index**_**n**_ **infected (AI**_**n**_**+)**	0	83.6	90.2	Reference		
	>0	16.2	9.8	1.77	[0.96–3.28]	0.067
**Pupae per House Index (PHI**_**n**_**)**	0	41.6	38.7	Reference		
	>0	58.4	61.3	0.83	[0.54–1.28]	0.397

### Entomological collections and indices

**Fig 4:**
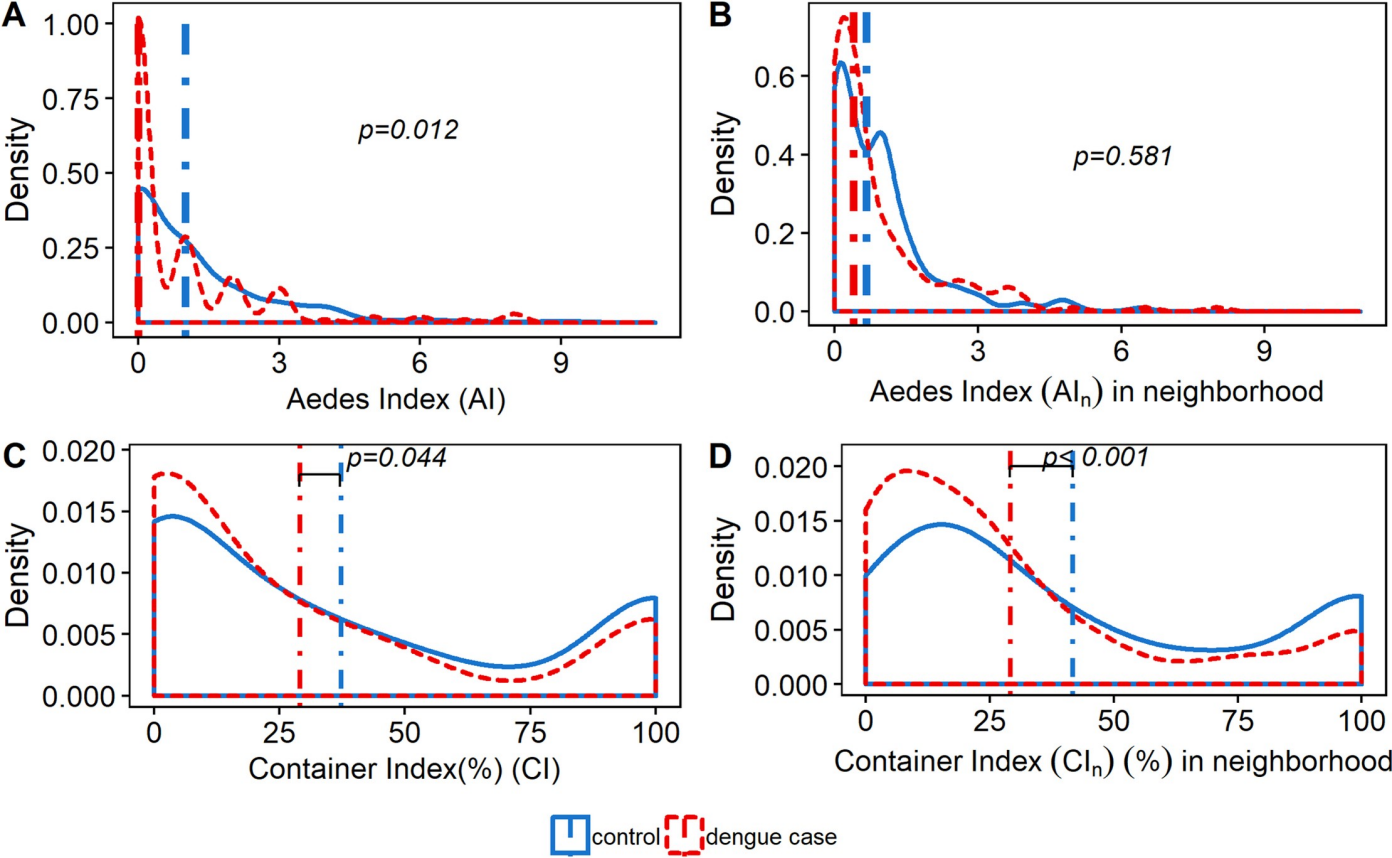
**Distribution of adult and immature *Aedes* indices in dengue case (red line) and control (blue line) houses.** Probability density distribution plots of *Aedes* Index (AI) at the patient house (**A**) and at the neighborhood level (including patient house) (**B**); and of the Container Index (CI) at the patient house **(C)** and at the neighborhood level (including patient house) **(D)**. The blue and red vertical lines in A and B represent the median *Aedes* indices in control and dengue case house, respectively. The blue and red vertical lines in C and D represent the mean container indices in control and dengue case houses, respectively. P-values were calculated using univariable logistic regression.

Entomological collections were carried in 1,487 households, of which 377 were patients houses and 1,110 surrounding houses (mean 3.94 houses per individual recruited). From 5,185 wet containers inspected, 1,230 (23.7%) were positive for immature *Aedes* stages, accounting for a total of 8,404 larval instars and 2,172 pupae. A total of 3,125 adult male and female *Aedes* were collected, the vast majority being *Ae*. *aegypti* (99.0%) and only 32 *Ae*. *albopictus* collected. Among the 1,224 females *Aedes* (39.2% of the total *Aedes* collected), 953 (77.8%) were collected indoors. Apart from the DENV-infected *Aedes* indices (AI+ and AI_n_+), all entomological indices had higher values in control houses than in case houses (Table 2), regardless of including the patient house with or without the neighboring houses. The *Aedes* Index, AI, (which includes both indoor and outdoor adult collections) was positive (i.e., at least one *Aedes* collected) in 38.7% of the case houses and in 51.5% of the control houses (Fig 4A). Moreover, the presence of *Aedes* was associated with lower odds of dengue (OR: 0.59, 95% CI: 0.39–0.89, p = 0.012). The *Aedes* Index indoor, AI_in was positive in 38.4% and 47.1% of the case and control houses, respectively. Similar to the AI, a positive AI_in was also associated with lower odds of dengue (OR: 0.53, 95% CI: 0.35–0.81, p = 0.003). Only the female *Aedes* infected, AI+ appears to be associated with increased dengue odds (OR: 2.48, 95% CI: 0.97–6.28, p = 0.056). The pupal indices, PPI and PHI, were not significantly different between case and control houses. Accounting only for the patient’s house (excluding neighbors), the Container Index was associated with the case/control status of houses, with a higher CI in the control than in the case houses (p = 0.044) (Fig 4C).

Only the *Aedes* infected index, AI_n_+ of mosquitoes collected in neighborhoods appears to be associated with higher odds of having a dengue case in the patient house, although the association was not statistically significant (OR: 1.77, 95%CI: 0.96–3.28, p = 0.067). Larval indices, CI_n_, BI and HI were negatively associated with dengue infections (p<0.001, p<0.001 and p = 0.002 respectively, Fig 4D). Likewise, the neighborhood adult *Aedes* indices (AI_n_ and AI_n__in) were higher in control households (Fig 4B). The presence of *Aedes* female (AI_n_), the presence of female *Aedes* indoors (AI_n__in), or the presence of *Aedes* pupae (PHI_n_) in the neighborhood were not significantly associated with dengue infection risk.

### Multivariable analysis of dengue fever occurrence

Using multivariable analysis, only a few entomological indices at the house level, compared to individual and household characteristics, were associated with dengue risk (Table 3). Individuals aged between 10 and 14 years and between 15 and 25 years had a higher odds of dengue infection than older adults (OR: 4.45, 95% CI: 2.14–9.24, p<0.001; OR: 2.88, 95% CI: 1.27–6.55, p = 0.012 respectively). Interestingly, younger children appeared to have similar odds as older adults, although with a wide confidence interval (OR: 1.05, 95% CI: 0.51–2.67, p = 0.707). Having an indoor workplace tended to higher odds than working outdoors (OR: 1.78, 95% CI: 0.94–3.36, p = 0.077). The type of house was also associated with dengue risk: living in a two-floor house had higher odds of dengue relative to a single floor dwelling (OR: 2.11, 95% CI: 1.21–3.69, p = 0.009). The presence of eaves gaps in the house was associated with lower odds of dengue (OR: 0.40, 95% CI: 0.23–0.68, p<0.001). The application of adult vector control methods was associated with higher odds of dengue (OR: 3.73, 95% CI: 1.19–11.7, p = 0.024). The presence of adult female *Aedes* inside the patient’s house was associated with lower odds of dengue (OR: 0.50, 95% CI: 0.19–0.73, p = 0.003). On the other hand, the presence of DENV-infected *Aedes* was associated with 4.20-fold higher odds of dengue infection compared to no infected mosquitoes present (OR: 4.20, 95% CI: 1.29–13.8, p = 0.018). In addition, the Container Index at the neighborhood level seemed associated with lower odds of dengue with OR of 0.93 per 10% increase (95% CI: 0.86–1.01, p = 0.089).

**Table 3:** Multivariable analysis of risk factors associated with dengue. Odds ratios (OR) were calculated by multivariable logistic regression and confidence intervals calculated using Wald’s statistics. Odds ratio in bold text were significant at p<0.05.

		OR		95% CI	p-value
**Age groups**	> 25 years old	Reference			
	15 to 25 years old	**2.88**		[1.27–6.55]	0.012
	10 to 14 years old	**4.45**		[2.14–9.24]	<0.001
	5 to 9 years old	1.05		[0.36–2.37]	0.899
**Location of workplace**	Outdoors	Reference			
	Indoors	1.78		[0.94–3.36]	0.077
	Both indoors and outdoors	0.70		[0.36–1.35]	0.281
**Travel within 3 months**	No	Reference			
	Yes	0.48		[0.20–1.15]	0.101
**Type of house**	One floor, one family	Reference			
	Two floors, one family	**2.11**		[1.21–3.69]	0.009
	Other	2.07		[0.61–6.99]	0.242
**Eaves gaps**	No	Reference			
	Yes	**0.40**		[0.23–0.68]	0.001
**Mosquito control**	None	Reference			
	Yes, against larvae	1.13		[0.44–2.89]	0.800
	Yes, against adult	**3.73**		[1.19–11.7]	0.024
	Yes, against both larvae and adult	0.63		[0.27–1.44]	0.272
***Aedes* Index indoor (AI_in)**	0	Reference			
	>0	**0.50**		[0.28–0.87]	0.014
***Aedes* Index infected (AI +)**	0	Reference			
	>0	**4.20**	[1.29–13.8]		0.018
**Neighborhood level**					
**Container**_**n**_ **Index CI**_**n**_ **(per 10% increase)**		0.93	[0.86–1.01]		0.089

## Discussion

In this hospital-based case-control study, we found that patient age, two-floor houses, application of adult vector control and the presence of DENV-infected *Aedes* were associated with higher odds of dengue. Interestingly, the presence of eave gaps in the house and the presence of female *Aedes* indoors were associated with lower odds of dengue. While dengue typically has had a greater impact on younger children, we found that individuals aged between 10 and 25 years-old were at higher risk relative to those either younger and older. This trend was also observed in several recent studies conducted in Thailand, Malaysia, and the Philippines [5, 43, 57, 58]. The increase in average age of infection may result from a change in demographic structure such as a decrease in birth rates or death rates [59, 60], leading to a lower proportion of naïve individuals or possibly a greater longevity of immune individuals in the population.

In northeastern Thailand, indoor workplaces are not always well protected against dengue mosquitoes, (e.g., shops lacking hard-wall storefronts, breeding container habitats within the building). *Aedes aegypti*, the main DENV vector in Thailand, is well adapted to human dwellings and their immediate surroundings. This day-biting mosquito typically feeds on multiple human hosts during each gonotrophic cycle, and usually rests indoors protected from more extreme outdoor elements [9]. This might explain the higher risk of dengue for individuals working indoors suggested in the current study. In contrast to other studies [61, 62], our results suggested that traveling outside the resident district during the previous three months was negatively associated with dengue risk (Table 1). Studies in Thailand have shown that dengue incidence is commonly spatially clustered [63, 64] and infection risk can be highly focal; thus moving out of the study areas might have exposed travelers to differential risks (higher or lower) of dengue transmission. Additional information to clarify areas traveled to, duration of trips, purpose, and the characterization of who is travelling might help resolve the negative association between dengue risk and travel seen in our study. Other individual characteristics were not informative for dengue risk using the multivariable model.

Our entomological findings showed that only the infected *Aedes* index at the household level (AI+) was positively associated with dengue infection, with more DENV positive females *Aedes* collected in case houses than in controls. A similar observation was found at the neighborhood level however not significant. In total, about 13% of the sampled neighboring households (including neighborhood and patient house) had DENV-positive female *Aedes*: 16% of the case neighboring households and about 10% of the control neighboring households. When focusing on the patient's houses specifically, approximately 3% of the control houses and 8% of the case houses had DENV-infected *Aedes*. The high proportion of DENV-infected *Aedes* demonstrates hyperendemicity conditions of dengue in northeastern Thailand [43]. In this study, determining the actual location of dengue case transmission is not possible. There is the possibility that the high proportion of DENV infected *Aedes* in case households was a result of DENV transmission from infected humans to the vectors present in the vicinity (i.e., not mosquito to human). For this study, vector infestation was measured only at the household level, thus recognizing that transmission could have happened elsewhere such as at schools or workplaces [65]. In Thailand, Ratanawong et al. [65] demonstrated the clustering of dengue cases among schools and among classrooms within schools, highlighting the importance of dengue transmission outside the home.

On the other hand, adult *Aedes* abundance in the household was negatively correlated with dengue with more *Aedes* found in control households than in houses with a recent dengue case. This counterintuitive association could be explained by potentially higher attention to mosquito control following onset of dengue symptoms in the case household, which would reduce vector infestation. Our results support this assumption as the associations between the *Aedes* Index indoor (AI_in) (Table 2), the mosquito control activities (Table 1) and the dengue risk were strengthened when adjusted for other variables (Table 3).

At the individual level, controls were more likely to have a high human immune response to *Aedes* salivary proteins than dengue cases, which correlate well with the higher abundance of *Aedes* adults in controls houses compared to case houses. This suggests that low responders actually received fewer *Aedes* bites than high responders, an observation previously shown in Benin [52]. Nevertheless, neither the adult abundance in the household nor the level of human exposure to *Aedes* mosquito bites were correlated with higher transmission risk. This can be explained by the fact that dengue virus transmission is complex and varies through time and space, and the relationship between vector density/aggressiveness and risk of human infection is not static. In addition, antibody response to *Aedes* saliva was positively correlated with IgG dengue immunity (S3 Table). Altogether, our data suggested that individuals with high exposure to *Aedes* have less odds of being dengue positive than individuals with lower exposure. However, the association of dengue IgG and antibodies to *Aedes* saliva with recent dengue infection was not strong enough to remain in the final multivariable model. The results of this study should be viewed with caution as the immune response reflects the overall exposure to *Aedes* bites in the previous weeks and not necessarily at the time of transmission. Additional longitudinal studies, including all inhabitants from each house, irrespective of dengue infection status, might better assess the association between exposure to *Aedes* bites and risk of dengue.

As in other dengue endemic countries, vector surveillance in Thailand focuses on immature stages, in particular, the standard larval indices (HI, BI, and CI). While a positive association between dengue cases and entomological indices was found in Cuba and Trinidad [21, 23] this has not been universally seen elsewhere [66, 67]. In our study, vector infestation indices based on immature stages (HI, BI, CI, and CI_n_) were all negatively associated statistically with dengue fever using univariable analysis. In other words, control households had more containers with immature *Aedes* than case households. However, this association was not statistically significant in the multivariable analysis except for CI_n_. Moreover, most (~90%) of the inspected houses had wet containers at the household and nearly half of the houses were positive for immature *Aedes*. Furthermore, most of households sampled in this study had index values above the minimum thresholds for dengue outbreak risk set by the Thai Ministry of Public Health [25]. During the study, the northeastern region of Thailand also experienced very low dengue incidence compared to the previous decade [40, 68].This study was conducted over a three-year period, thus capturing intra- and inter-epidemic dengue transmission in this northeastern region of Thailand. Dengue transmission in Thailand is highly seasonal with the highest incidence occurring during the wet season (May-October) [5]. This may account for the high proportion of houses with water-storage containers found positive for immature *Aedes*.

Other studies have found a higher risk of dengue transmission in poorer settings [69–71]. However, in our study, no such association was found (S4 Table). Nevertheless, household construction may play a role in transmission risk, wherein people living in two-floor houses appear to have had a greater risk for contracting dengue. Interestingly, in our study settings two-floor households were more commonly found among farmers (S5 Table). In addition, in rural two-floor houses, the lower one is often used for gatherings of family or community members, friends or neighbors [72], which may increase the risk of dengue [73]. The negative association between eaves gaps in houses and dengue risk appear counterintuitive (i.e., increased access for mosquitoes to enter a house). In central and southern Thailand, Brusich et al. [74] showed in rural settings, households with <25% eaves gaps have, overall, more mosquitoes indoors than those with 50% to 75% eaves gaps. Moreover, they reported that vector control activities were absent in houses with <25% eaves gaps and that bed nets were more systematically used in houses with >50% eaves gaps. However, the results from their study should be interpreted cautiously as it is based on few houses [74]. Nevertheless, the authors suggested that the presence of eaves gaps might result in a higher abundance of mosquitoes, which in turn, might induce more vector control activities by the household to reduce biting. However, in our study, no correlation was found between the presence of eaves gaps in the households and vector control methods used (S6 Table). Moreover, an apparent ‘protective’ effect by presence of eaves gaps on dengue risk might be explained by the location of productive breeding sites. Indeed, if the majority of container habitats are located indoors, eaves gaps can represent exit routes for the vectors [75].

We identified two previous case-control studies of dengue with similar designs, i.e. both cases and controls recruited in health facilities, with controls being “test-negative”: one in Singapore [76] and another in Malaysia [46]. The Malaysian study included two sets of controls: one test-negative and the other being hospitalized (inpatient) with no suspicion of dengue (“traditional” control). In their analysis, no risk factors were identified in the test-negative controls, although the number of them was small (28). The authors suggest that test-negative studies could be subject to bias resulting from misclassification of dengue status due to imperfect diagnostic tests. In Singapore, the controls which were either DENV-PCR negative or had no evidence of seroconversion on follow-up, analysis found no associations between dengue risk and house construction, travel, working outdoors or indoors, or self-reported history of mosquito bites [76]. In the current study, misclassification of dengue infection is unlikely to be a major problem because all controls were PCR-negative and all but 12 (being RDT NS1 antigen and/or IgM positive only) of the 184 cases were DENV-PCR positive (Fig 1). However, we cannot rule out that our controls were infected with other *Aedes*-borne viruses such as chikungunya or Zika, and thereby biasing our assessment of the entomological risk factors. Chikungunya fever incidence was extremely low during the 2016–2017 period, with a total of 18 and 10 cases, in 2017 and 2016 respectively but increased to around 3600 cases in 2018, although the epidemic was centered in southern Thailand [40, 77, 78]. In addition, CHIKV was detected among eight patients out of 161 tested in the period 2016–2017 in our study participants [79]. Regarding Zika infection, a recent study demonstrated the circulation of the virus, at low incidence, in Thailand for years [80]. Indeed, the Bureau of Epidemiology of Thailand reported a cumulative number of 1,612 Zika cases for the period 2016–2017, while more than 118,000 dengue cases were reported during the same period [77, 78, 81]. Although potential dengue cases have similar febrile symptoms as potential controls (with other conditions), any difference in health-seeking behavior between them may have also biased the results [82]. Thailand has a universal health coverage program that allows people access to equitable and effective healthcare in primary care centers located in each subdistrict [83, 84]. Therefore, by recruiting patients at the main district hospitals, we feasibly captured a high proportion of the febrile patients, including children, living in the area.

Our study presented some further limitations in terms of generalizability. During the study period, dengue incidence was lower than expected, despite the high percentage of DENV-infected *Aedes* found in our study, the 173 cases were obtained only after extending the original study period and coverage area. This may suggest a high proportion of immune individuals. In Thailand, all four serotypes are endemic, dengue vector species are widespread, and a high percentage of DENV infected vectors may lead to a high proportion of dengue-immune individuals in the population, lessening dengue incidence. The relationship between entomological risk factors and dengue may vary according to the extent of serotype-specific immunity in the population and this, in turn, may vary between high and low incidence years and the predominant virus serotype(s) in circulation. Indeed, during 2017–2018, the main DENV serotype circulating among dengue cases was DENV-1, with an increased prevalence compared with the previous six years, while the prevalence of DENV-4 was lower than previous years. In addition, DENV-3 was the prevalent serotype between 2013 and 2015 accounting for approximately 30% of the dengue cases [40, 85–87]. As a result, caution is advised with drawing associations of risk with entomological thresholds as they depend on the immune status of the human population under study [22, 24, 88].

Another limitation is that we focused on household entomological indices, yet the transmission could have occurred in other locations and at other times, especially for many children who spend most of their daytime hours at school. Including workplaces, schools and community centers where people gather might be helpful for understanding dengue transmission risk outside the household setting [65]. In this study, information on these other locations is limited and indirect. Most dengue case-control studies focused on the epidemiological risk factors associated with higher severity of dengue disease, while fewer have investigated the role of entomological factors. Moreover, the majority of those studies used immature *Aedes* indices to assess the infestation level (density) in the study area [61, 62]. Nevertheless, a study in Sao Paulo, Brazil demonstrated a strong association between numbers of female *Aedes* collected over a fortnight and dengue incidence [89]. Their findings were obtained after the re-introduction of DENV serotype 3, to which the majority of the population were susceptible, thus facilitating the assessment of entomological risk factors.

The retrospective case-control design means the temporal sequence of events cannot be determined with accuracy. In particular, entomological and immunological data were collected following patient recruitment. Indeed, symptoms of dengue fever can appear as quickly as a few days after DENV transmission (typically incubation period between 4–7, up to 14 days), delaying the recruitment of patients and therefore the entomological collections. This temporal disconnection between acquiring an infection to time of presenting illness and testing (i.e., identification of a case) may greatly affect attempts to link transmission with actual epidemiological conditions many days prior. Although speculative, the occurrence of a dengue case might plausibly prompt householders to reduce adult vector density, while the remaining mosquitoes may retain a higher prevalence of infection when the case is detected. A longitudinal, prospective study design might better assess the impact of entomological indices on dengue transmission risk in northeastern Thailand.

Our case-control study in northeastern Thailand highlights the complex relationship between *Aedes* vectors, socio-economic factors, and dengue transmission risk. The presence of DENV-infected *Aedes* was associated with higher odds of dengue infection. Our findings support the rationale of monitoring DENV in adult *Aedes* vectors resting in and near houses to assess risk of dengue transmission [90–92] and to develop early warning indicators for dengue outbreak prevention [93]. Although adult surveillance holds promise as an additional, if not more informative, *Aedes*-borne disease risk indicator, further work is needed investigating simple, inexpensive passive sampling tools to make this a feasible strategy. The results also suggest that monitoring dengue vector abundance alone, in particular immature-stage indices, may not be accurate enough to identify households at heightened risk of dengue infection.

## Supporting information

S1 STROBE Statement Checklist of items that should be included in reports of case-control studies. (DOCX)

S1 Table Variables definition. (DOCX)

S2 Table List of components used for the SES calculation according to Vyas and Kamaranayanke, using varimax rotation. (DOCX)

S3 Table Association between antibody response to *Aedes* saliva in household inhabitants and presence of dengue IgG. Odds ratios obtained by logistic univariable regression and confidence intervals (95% CI) by Wald’s statistics.
(DOCX)

S4 Table Association between household characteristics with dengue risk on a subset of the dataset (n = 252 houses, including 153 controls and 99 dengue cases). Statistical analysis was conducted in R 3.5.1 software using logistic univariate regression and 95% confidence intervals (95% CI) were calculated using Wald statistics.
(DOCX)

S5 Table Association between farming being the main source of income and household type in northeastern Thailand. Statistical analysis was conducted in R software 3.5.1 using a logistic binomial regression. 95% Confidence Intervals (95% CI) were calculated using Wald statistics. Odds ratio in bold are significant at p<0.05.
(DOCX)

S6 Table Differences in types of vector control activities in households with or without eaves gaps in northeastern Thailand, June 2016 and August 2019. Statistical analysis was conducted in R 3.5.1 software using chi-square (χ2) test of independence for categorical variables.
(DOCX)
