# Supplementary material for: Complex relationships between Aedes vectors, socio-economics and dengue transmission—Lessons learned from a case-control study in northeastern Thailand
Source: PLoS Negl Trop Dis. 2020 Oct 1;14(10):e0008703. doi: 10.1371/journal.pntd.0008703 (PMC7553337; doi:10.1371/journal.pntd.0008703)
Supplement: S1 Table — (DOCX) [file pntd.0008703.s002.docx]

| **Variable** | **Definition / Formula** | ***Aedes* life stage** |
| --- | --- | --- |
| **Individual level** | |  |
| MEI | MEI= ∆OD-TR  IgG response to Nterm 34 kDa salivary peptide | Adult |
| **Household level (patient house)** | |  |
| CI (%) | (No. positive container/ total no of wet container) x 100 | Larvae & Pupae |
| AI | No. of adult female *Aedes* collected | Adult |
| AI_in | No. of adult female *Aedes* collected indoors (only) | Adult |
| AI+ | Proportion of infected females *Aedes* in the patient house | Adult |
| PHI | No. of pupae collected at the patient house | Pupae |
| PPI | No. of pupae collected per person at the patient house | Pupae |
| **Neighborhood level** | |  |
| CI_n_ (%) | (No. positive container/ total no. of wet container) x 100 | Larvae & Pupae |
| HI (%) | (No. positive house/ no. households visited) x 100 | Larvae & Pupae |
| BI | No. positive container x100/ No. containers inspected / no. house visited | Larvae & Pupae |
| AI_n_ | No. of adult female *Aedes* collected/ no. households visited | Adult |
| AI_n__in | No of adult female *Aedes* collected indoor/ no. households visited | Adult |
| AI_n_+ | Proportion of infected adult female *Aedes*/ no. houses visited | Adult |
| PHI_n_ | No. of pupae collected/ no. houses visited | Pupae |

**S1 Table.** Variables definition
