# Supplementary material for: Complex relationships between Aedes vectors, socio-economics and dengue transmission—Lessons learned from a case-control study in northeastern Thailand
Source: PLoS Negl Trop Dis. 2020 Oct 1;14(10):e0008703. doi: 10.1371/journal.pntd.0008703 (PMC7553337; doi:10.1371/journal.pntd.0008703)
Supplement: S2 Table — (DOCX) [file pntd.0008703.s003.docx]

**S2 Table.** List of components used for the Socio-Economical Status calculation according to Vyas and Kamaranayanke, using varimax rotation.

|  | **Comp.1** | **Comp.2** | **Comp.3** | **Comp.4** | **Comp.5** | **Comp.6** | **Comp.7** | **Comp.8** | **Comp.9** | **Comp.10** | **Comp.11** | **Comp.12** | **Comp.13** | **Comp.14** | **Comp.15** | **Comp.16** |
| --- | --- | --- | --- | --- | --- | --- | --- | --- | --- | --- | --- | --- | --- | --- | --- | --- |
| Air Conditioner | 0.40 | 0.13 | 0.19 | 0.02 | 0.17 | 0.25 | 0.13 | 0.07 | 0.18 | 0.00 | 0.27 | 0.07 | 0.26 | 0.66 | 0.10 | 0.23 |
| Computer | 0.41 | 0.12 | 0.09 | 0.14 | 0.01 | -0.05 | -0.18 | -0.15 | 0.18 | 0.22 | -0.11 | -0.54 | 0.27 | -0.36 | -0.24 | 0.29 |
| Microwave | 0.44 | 0.11 | 0.14 | 0.15 | -0.05 | -0.14 | -0.10 | 0.01 | 0.08 | 0.05 | -0.43 | 0.04 | -0.04 | 0.06 | 0.40 | -0.60 |
| Mobile phone | 0.01 | -0.37 | 0.25 | 0.39 | 0.44 | -0.02 | -0.09 | -0.04 | -0.14 | -0.27 | 0.20 | -0.16 | -0.03 | 0.07 | -0.38 | -0.37 |
| Motorcycle | 0.05 | -0.37 | -0.33 | -0.34 | 0.05 | -0.21 | 0.20 | 0.26 | 0.09 | -0.22 | -0.29 | -0.50 | -0.03 | 0.30 | 0.01 | 0.03 |
| Oven | 0.33 | -0.09 | 0.09 | -0.27 | 0.14 | -0.46 | -0.05 | -0.20 | 0.23 | 0.05 | 0.01 | 0.36 | -0.47 | 0.02 | -0.30 | 0.17 |
| Personal car | 0.35 | 0.10 | -0.12 | 0.10 | -0.01 | 0.18 | -0.09 | 0.51 | -0.37 | 0.05 | 0.22 | -0.14 | -0.54 | -0.13 | 0.08 | 0.12 |
| Pickup | 0.12 | -0.23 | 0.48 | -0.28 | -0.20 | -0.13 | 0.36 | -0.21 | -0.17 | -0.11 | 0.37 | -0.21 | 0.00 | -0.22 | 0.36 | -0.02 |
| Refrigerator | 0.14 | -0.39 | -0.20 | -0.07 | -0.04 | 0.16 | 0.11 | -0.28 | -0.41 | 0.66 | -0.06 | 0.06 | 0.06 | 0.16 | -0.12 | -0.11 |
| Smart Phone | 0.20 | 0.38 | -0.35 | -0.13 | -0.27 | -0.10 | 0.28 | -0.04 | -0.02 | -0.13 | 0.35 | -0.03 | 0.14 | -0.01 | -0.40 | -0.43 |
| TV | 0.13 | -0.24 | -0.32 | 0.08 | -0.10 | 0.52 | -0.03 | -0.40 | 0.43 | -0.20 | 0.11 | -0.05 | -0.30 | -0.11 | 0.14 | -0.06 |
| Van | 0.08 | 0.03 | -0.04 | -0.49 | 0.02 | 0.08 | -0.72 | -0.18 | -0.29 | -0.24 | 0.09 | -0.04 | 0.15 | 0.09 | 0.05 | -0.06 |
| VCR/CD/ DVD | 0.19 | -0.03 | -0.40 | 0.40 | 0.06 | -0.35 | 0.08 | -0.29 | -0.34 | -0.32 | 0.00 | 0.15 | 0.16 | -0.01 | 0.26 | 0.31 |
| Washing machine | 0.29 | -0.35 | -0.04 | -0.16 | 0.08 | 0.19 | 0.09 | 0.38 | 0.06 | -0.16 | -0.08 | 0.44 | 0.40 | -0.42 | -0.08 | 0.00 |
| 2Wheel Tractor | -0.06 | -0.34 | -0.14 | 0.21 | -0.32 | -0.35 | -0.36 | 0.25 | 0.31 | 0.24 | 0.45 | 0.02 | 0.14 | 0.08 | 0.16 | -0.06 |
| 4Wheel Tractor | 0.09 | -0.16 | 0.26 | 0.19 | -0.71 | 0.09 | -0.06 | 0.01 | -0.14 | -0.26 | -0.27 | 0.08 | -0.05 | 0.21 | -0.32 | 0.15 |
|  |  |  |  |  |  |  |  |  |  |  |  |  |  |  |  |  |
| **Eigenvalue** | 2.64 | 1.81 | 1.35 | 1.26 | 1.13 | 1.03 | 0.96 | 0.89 | 0.82 | 0.76 | 0.67 | 0.64 | 0.57 | 0.53 | 0.47 | 0.45 |
| **Standard deviation** | 1.63 | 1.35 | 1.16 | 1.12 | 1.06 | 1.01 | 0.98 | 0.95 | 0.91 | 0.87 | 0.82 | 0.80 | 0.76 | 0.73 | 0.69 | 0.67 |
| **Proportion of Variance** | 0.17 | 0.11 | 0.08 | 0.08 | 0.07 | 0.06 | 0.06 | 0.06 | 0.05 | 0.05 | 0.04 | 0.04 | 0.04 | 0.03 | 0.03 | 0.03 |
| **Cumulative Proportion** | 0.17 | 0.28 | 0.36 | 0.44 | 0.51 | 0.58 | 0.64 | 0.69 | 0.74 | 0.79 | 0.83 | 0.87 | 0.91 | 0.94 | 0.97 | 1.00 |
