# Supplementary material for: Complex relationships between Aedes vectors, socio-economics and dengue transmission—Lessons learned from a case-control study in northeastern Thailand
Source: PLoS Negl Trop Dis. 2020 Oct 1;14(10):e0008703. doi: 10.1371/journal.pntd.0008703 (PMC7553337; doi:10.1371/journal.pntd.0008703)
Supplement: S3 Table — Odds ratios obtained by logistic univariable regression and confidence intervals (95% CI) by Wald’s statistics. (DOCX) [file pntd.0008703.s004.docx]

**S3 Table.** Association between antibody response to *Aedes* saliva in household inhabitants and being positive for dengue IgG. Odds ratios obtained by logistic univariable regression and confidence intervals (95% CI) by Wald’s statistics.

|  | | **Odds Ratio** | **95% CI** | | **p-value** |
| --- | --- | --- | --- | --- | --- |
| **Mosquito Exposure Index (MEI)** | Non responder | Reference |  |  |  |
|  | Low responder | 3.00 | [0.65-13.8] | | 0.15 |
|  | Medium responder | 4.08 | [0.90-18.4] | | 0.07 |
|  | High responder | 4.03 | [0.89-18.1] | | 0.07 |
