# Supplementary material for: Complex relationships between Aedes vectors, socio-economics and dengue transmission—Lessons learned from a case-control study in northeastern Thailand
Source: PLoS Negl Trop Dis. 2020 Oct 1;14(10):e0008703. doi: 10.1371/journal.pntd.0008703 (PMC7553337; doi:10.1371/journal.pntd.0008703)
Supplement: S4 Table — Statistical analysis was conducted in R 3.5.1 software using logistic univariate regression and 95% confidence intervals (95% CI) were calculated using Wald statistics. (DOCX) [file pntd.0008703.s005.docx]

**S4 Table.** Association between household characteristics with dengue risk on a subset of the dataset (n=252 houses, including 153 controls and 99 dengue cases). Statistical analysis was conducted in R 3.5.1 software using logistic univariate regression and 95% confidence intervals (95% CI) were calculated using Wald statistics.

| **Variable** | **Level** | **Odds ratio (OR)** | **95% CI** | **p-value** |
| --- | --- | --- | --- | --- |
| **Household characteristics** | | | | |
| **Income category (in Thai bath)** | >50,000 | Reference |  |  |
|  | 10,001-50,000 | 0.9 | [0.4-1.6] | 0.665 |
|  | 0-10,000 | 1.4 | [0.8-2.6] | 0.254 |
| **Socioeconomic status** | High | Reference |  |  |
|  | Intermediate | 1.3 | [0.7-2.5] | 0.348 |
|  | Low | 0.9 | [0.5-1.6] | 0.630 |
| **Wall types** | Plastered | Reference |  |  |
|  | Cement/Bricks | 0.7 | [0.4-1.4] | 0.393 |
|  | Other | 1.7 | [0.7-4.2] | 0.257 |
| **Floor types** | Tiles | Reference |  |  |
|  | Cement | 0.7 | [0.4-1.2] | 0.175 |
|  | Other | 1.1 | [0.4-2.9] | 0.783 |
| **Roof types** | Ceramic | Reference |  |  |
|  | Metal | 0.5 | [0.1-2.7] | 0.430 |
|  | Other | 1.0 | [0.5-1.8] | 0.986 |
| **Ceiling types** | No ceiling | Reference |  |  |
|  | Wooden boards, fitted | 1.1 | [0.4-3.2] | 0.885 |
|  | Wooden boards, fitted | 0.2 | [0.02-1.5] | 0.109 |
|  | Gypsum | 1.2 | [0.7-2.2] | 0.503 |
|  | Others | 2.4 | [0.7-9.0] | 0.182 |
| **Location of toilet** | Indoor | Reference |  |  |
|  | Outdoor | 0.7 | [0.4-1.3] | 0.265 |
| **Eaves gap** | No | Reference |  |  |
|  | Yes | 0.7 | [0.4-1.1] | 0.135 |
| **House types** | One floor, one family | Reference |  |  |
|  | Two floor, one family | 1.4 | [0.8-2.3] | 0.263 |
|  | Others | 1.3 | [0.5-3.7] | 0.624 |
| **Mosquito prevention technique** | | | | |
| **Mosquito control** | Against larvae | Reference |  |  |
|  | Against adult | 1.2 | [0.4-3.4] | 0.730 |
|  | Against both adult and larvae | 0.5 | [0.3-1.1] | 0.082 |
|  | No control | 1.1 | [0.4-3.0] | 0.867 |
| **Spray use** | Yes | Reference |  |  |
|  | No | 0.7 | [0.4-1.2] | 0.212 |
| **Window screen** | Yes | Reference |  |  |
|  | No | 1.1 | [0.5-2.0] | 0.863 |
| **Outdoor fogging** | Yes | Reference |  |  |
|  | No | 1.5 | [0.8-2.5] | 0.196 |
| **Temephos use** | Yes | Reference |  |  |
|  | No | 1.5 | [0.8-2.6] | 0.189 |
| **Frequency of temephos** | No | Reference |  |  |
|  | Yes, daily | 0.3 | [0.06-1.5] | 0.137 |
|  | Yes, weekly | 0.8 | [0.4-1.7] | 0.614 |
|  | Yes, monthly | 0.7 | [0.3-1.4] | 0.265 |
|  | Less frequently | 0.6 | [0.3-1.3] | 0.209 |
| **Larvae control** | Yes | Reference |  |  |
|  | No | 1.5 | [0.4-4.9] | 0.521 |
| **Bed net use** | Yes | Reference |  |  |
|  | No | 1.1 | [0.6-2.1] | 0.757 |
| **Container cleaning** | Yes | Reference |  |  |
|  | No | 0.9 | [0.5-1.7] | 0.850 |
