# Supplementary material for: Complex relationships between Aedes vectors, socio-economics and dengue transmission—Lessons learned from a case-control study in northeastern Thailand
Source: PLoS Negl Trop Dis. 2020 Oct 1;14(10):e0008703. doi: 10.1371/journal.pntd.0008703 (PMC7553337; doi:10.1371/journal.pntd.0008703)
Supplement: S5 Table — Statistical analysis was conducted in R software 3.5.1 using a logistic binomial regression. 95% Confidence Intervals (95% CI) were calculated using Wald statistics. Odds ratio in bold are significant at p<0.05. (DOCX) [file pntd.0008703.s006.docx]

**S5 Table.** Association between farming being the main source of income and household type in northeastern Thailand. Statistical analysis was conducted in R software 3.5.1 using a logistic binomial regression. 95% Confidence Intervals (95% CI) were calculated using Wald statistics. Odds ratio in bold are significant at p<0.05.

|  |  | **Odds Ratio** | **95% CI** | **p-value** |
| --- | --- | --- | --- | --- |
| **Type House** | One-floor, one family | Reference |  |  |
|  | Two-floor, one family | **2.31** | [1.46-3.66] | <.001 |
|  | Others | **0.25** | [0.07-0.87] | 0.029 |
