# Supplementary material for: Complex relationships between Aedes vectors, socio-economics and dengue transmission—Lessons learned from a case-control study in northeastern Thailand
Source: PLoS Negl Trop Dis. 2020 Oct 1;14(10):e0008703. doi: 10.1371/journal.pntd.0008703 (PMC7553337; doi:10.1371/journal.pntd.0008703)
Supplement: S6 Table — Statistical analysis was conducted in R 3.5.1 software using chi-square (χ2) test of independence for categorical variables. (DOCX) [file pntd.0008703.s007.docx]

|  | | **Eaves gaps** | | | | | **p-value** (χ^2^) |
| --- | --- | --- | --- | --- | --- | --- | --- |
|  |  | No | | Yes | | |  |
|  |  | N | (%) | n | | (%) |  |
| **Vector control** | Yes, against larvae | 45 | (21.6%) | 41 | (27.7%) | | 0.5909 |
|  | Yes, against adult mosquito | 23 | (11.1%) | 16 | (10.8%) | |  |
|  | Yes, against both adult and larvae | 118 | (56.7%) | 75 | (50.7%) | |  |
|  | No | 22 | (10.6%) | 16 | (10.8%) | |  |

**S6 Table.** Differences in types of vector control activities in households with or without eaves gaps in northeastern Thailand, June 2016 and August 2019. Statistical analysis was conducted in R 3.5.1 software using chi-square (χ^2^) test of independence for categorical variables.
